# Influence of Clogging and Unbound Base Layer Properties on Pervious Concrete Drainage Characteristics

**DOI:** 10.3390/ma13112455

**Published:** 2020-05-28

**Authors:** Ivana Barišić, Ivanka Netinger Grubeša, Tihomir Dokšanović, Matija Zvonarić

**Affiliations:** Faculty of Civil Engineering and Architecture Osijek, Josip Juraj Strossmayer University of Osijek, Vladimira Preloga 3, 31000 Osijek, Croatia; nivanka@gfos.hr (I.N.G.); tdoksanovic@gfos.hr (T.D.); mzvonaric@gfos.hr (M.Z.)

**Keywords:** pervious concrete, urban pavement, drainage, clogging, pavement system, unbound base layer

## Abstract

This paper aims to assess the influence of clogging on paving material (pervious concrete) drainage characteristics as well as the influence of the properties of an unbound base layer on drainage characteristics of the whole paving system. The clogging influence has been studied measuring the drainage characteristics on pervious concrete flags before and after their clogging, according to ASTM C1701-09. Additionally, the drainage characteristics of uncontaminated pervious concrete as a paving material was assessed using the falling head method. To assess the influence of properties of an unbound base course (UBC) on drainage characteristics of the whole paving system, the unbound base layer was compacted in two different levels of compaction and the drainage characteristics were measured (according to ASTM C1701-09). It is concluded that pervious concrete prepared with a smaller aggregate fraction is more prone to clogging. Regarding the influence of UBC, it is important to find a balance between pervious concrete infiltration and UBC exfiltration rate, particularly in a case of pervious concrete flags made of coarse aggregate.

## 1. Introduction

Good drainage presents a necessity for quality road projects. This is a particularly important issue within highly populated areas or urban areas where good surface drainage presents an imperative for quality daily life. Past years of investigations have provided some new materials and technologies for effective drainage, and pervious pavements were emphasized as a green, sustainable paving solution. Such a pavement is typically considered as the surface course comprised of permeable block pavers, porous asphalt or pervious concrete, generally with the underlying bedding layer separated from the coarse aggregates beneath by geotextiles. As a dominant building material for many years, concrete is used for various applications which utilize its specific characteristics. Green paving with pervious concrete is accentuated by its ability to reduce runoff water, improve runoff water quality, reduce on-site noise level as well as relieve the heat island effect [1,2,3,4]. Even though it is comprised of the same basic components as standard concrete, the reduction or even absence of fine aggregate in pervious concrete mixture presents a possibility for natural aggregate preservation. This uniform composite made of coarse aggregate, cement and water, is designed to have high porosity (void content between 11% and 35% [5,6]) and permeability (typically about 2–6 mm/s [5,7]), but consequently relatively low strengths (compressive strength varies between 3.5 MPa and 28 MPa, and flexural strength varies between 1 MP and 3.8 MPa [7]). A large number of studies are oriented to improving its mechanical properties and preserving high porosity, mostly by using new additives and materials. For example, strength increase may be achieved by incorporating fine sand [8], but this reduces the porosity and permeability of pervious concrete. Using copper slag as coarse aggregate [9], nano-silica [10], polymer (latex) [11] or embedding geogrids can improve the strength and restrain cracking [4]. Elizondo-Martinez et al. [12] investigated the most common mixture design method, followed by most researchers and prescribed in ACI 522R-10, and concluded that it is possible to improve pervious concrete mechanical properties just by changing the mixture design method. On the other hand, enhancing some other important pavement wearing course parameters, such as resistance to sulphate attack and abrasion (by replacing cement with metakaolin [13]) or the micro-texture and skid resistance (by using corundum as fine aggregate [14]), may lead to a decrease in strength.

Permeability, as one of the main features of pervious concrete, is assessed with different laboratory and field methods. The most used ones are constant and falling head methods and the method described in standard ASTM C1701-09 [15], for in-placed pervious concrete pavement. Constant and falling head methods (CHM and FHM) are commonly used in soil mechanics and seem to be more familiar to the average civil engineer, while the ASTM C1701-09 method is the only one defined for use in pervious concrete testing. Comparing the results obtained by these three methods, it can be concluded that FHM presents the highest and ASTM C 1701-09 the lowest permeability values, although trends are the same [16] and all three tests have acceptable levels of uncertainty in comparison to sample-to-sample variation [17]. A falling head test is easier to operate than the constant head and it is recommended to conduct the tests at low head (water) levels [17], as rain events typically result in a small amount of water on the pavement surface. Also, a low head level (as in ASTM C1701-09) will give the most realistic results.

Pervious concrete has certain limiting usage factors, of which the most important ones are durability issues related to abrasion and freeze-thaw cycles resistance. Researchers have shown that increasing mechanical properties by different additives (latex [18], long macro fibres [19], silica fume with superplasticizers [20], tire chips and crumb rubber [2]) can lead to durability improvement. One of the main disadvantages of pervious concrete pavement is also its maintenance, which is oriented mainly to clogging prevention that can lead to permeability reduction. Numerous research works within the last few years [21,22,23,24] emphasize the importance of the clogging phenomenon on pervious concrete pavement performance. Clogging is caused by the interlocking of the pores with clogging particles and pore size definition can be useful in determining clogging potential [25], which is also related to the tortuosity of the connected porosity [26]. According to [27], the permeability considerably reduces due to clogging within the first five years of service. In [28] a mathematical model is proposed which could be developed to enable service life modelling of permeable concrete pavement. Additionally, a lot of research emphasizes the type of clogging agent [23,27,29]. By analysing the clogging phenomenon, it was concluded that sediment size distribution has a significant influence on clogging development. Coarse sands are more likely to cause clogging on the surface of pervious concrete elements, while fine sands will percolate into the depth [30]. Clay is much more damaging than sand [31], which is attributed to the plasticity of fine sediments that form clay flocks due to the Van der Waal forces [21].

While surface drainage is important in terms of traffic safety, underground drainage is essential for roadway stability and durability. This entails that it is important to look at the pavement system as a whole and, due to the inability to test under realistic conditions, to simulate in situ conditions as closely and accurately as possible in the laboratory. This paper aims to assess the influence of clogging on paving material (pervious concrete) drainage characteristics as well as the influence of the properties of the unbound base layer on drainage characteristics of the whole paving system. The clogging influence has been studied measuring the drainage characteristics on pervious concrete flags before and after their clogging, according to ASTM C1701-09. The clogging was simulated using a mixture of clay and sand as suggested in [32]. Additionally, the drainage characteristics of uncontaminated pervious concrete as a paving material were assessed using the falling head method. To assess the influence of properties of unbound base layer on drainage characteristics of the whole paving system, the unbound base layer was compacted in two different levels of compaction and the drainage characteristics were measured (according to ASTM C1701-09).

## 2. Materials and Methods

### 2.1. Materials Used, Pervious Concrete Sample Casting and Curing

**Figure 1:**
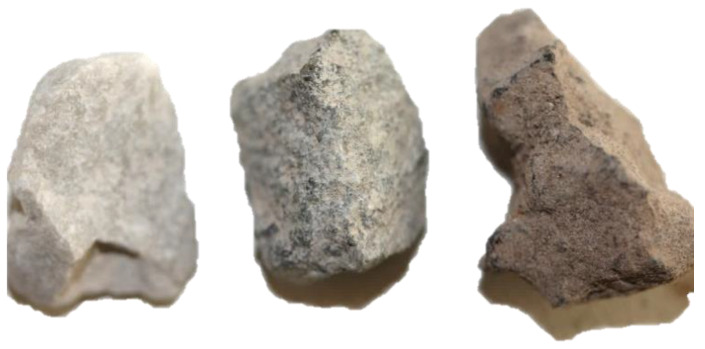
The appearance of the aggregate grain: dolomite, diabase and steel slag.

Pervious concrete mixtures with three different types of aggregates and different proportions of aggregate fractions were used within this study, accounting for 11 different pervious concrete mixtures in total (Table 1). Crushed dolomite and diabase stone, traditional construction materials in the Republic of Croatia were used as natural materials, and steel slag from a Croatian landfill near the town of Sisak was used as a substitute, waste aggregate material with significantly different surface textures (Figure 1). The relevant properties of the aggregates are given in [33] and [16]. As a fine fraction, sand from the Drava River (fraction 0–2 mm) and crushed diabase (fraction 0–2 mm) were used. The densities of the used crushed dolomite aggregate, diabase, steel slag, and sand were 2.75, 2.91, 3.21 and 2.65 kg/dm^3^, respectively, according to EN 1097-6 [34].

For all mixtures, the water to cement ratio (*w/c*) was 0.33, with tap water. The cement was CEM II/A-M(S-V) 42.5 N, per EN 197-1 [35], having a density of 3.0 kg/dm^3^ per EN 196-6 [36]. The cement content was 300 kg/m^3^ for all mixtures. Specimens of all concrete mixtures were cast with a compacting rod, by rodding 25 times. All specimens were extracted from moulds 24 h after casting and placed in a water tank for 27 days, at a temperature of 20 ± 5 °C, per EN 12390 2 [37].

### 2.2. Testing of Hardened Pervious Concrete Properties

At 28 days of age, the properties of three samples per mixture of hardened concrete were tested. Compressive strength was tested on cube specimens of 15 cm edge length, with a constant rate of loading of 0.5 MPa/s, per EN 12390-3 [38]. Flexural strength was tested on prism specimens with measurements 10 × 10 × 40 cm, by loading them with a constant rate of 0.05 MPa/s, per EN 12390-5 [39]. Total porosity was determined with the following methodology: (1) determination of dry and saturated surface dried mass of the specimen (weighed in water and air); (2) calculation of density and apparent density from parameters determined in (1); (3) calculation of solid material volume and isolated pore volume with corresponding basic formulas, with the assumption that the specimen total volume is a sum of solid material volume, isolated pore volume, and connected pore volume.

### 2.3. Testing of Drainage Characteristics of Pervious Concrete and Paving System

The falling head (FHM) method was used on small cylindrical samples 5 cm diameter and 10 cm in height on uncontaminated pervious concrete samples, and the standardized test method per ASTM C1701-09 [15] was used for testing the infiltration rate of pervious concrete before and after their clogging as well as the infiltration rate of the whole paving system (pervious concrete + unbound base layer). The FHM method is based on the time required for a certain water column to drop through the sample, determined by recording the time interval during this process. The standardized method described in ASTM C 1701-09 determines the field water-infiltration rate of in-place pervious concrete by recording the run-off time of a certain water volume through a defined area of porous concrete pavement. Within this research, pervious concrete flags measuring 50 × 50 × 5 cm were used for simulation of inbuilt pavement.

**Figure 2:**
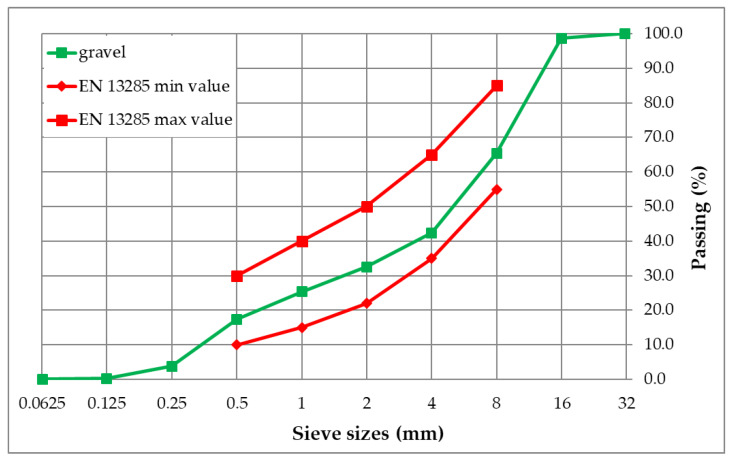
The grain size distribution of gravel used for the unbound base layer.

To simulate and consider realistic parameters for drainage characteristics valorisation of laboratory-made pervious concrete samples, a new test setup is devised. As the unbound base course (UBC), natural gravel is used with grain size distribution presented in Figure 2. Standard EN 13285 [40] was used for unbound mixture characterization.

**Figure 3:**
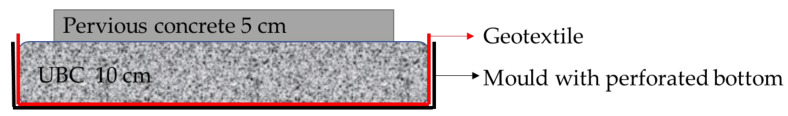
Test setup for pervious concrete-unbound base layer system drainage testing.

Maximum dry density and water content were determined by vibrating hammer according to standard EN 13286-4 [41]. The material was then inbuilt in two moulds with a vibrating hammer, controlling the mass of inbuilt material and calculating water content and density of unbound base layer after compaction. Characteristics of 10 cm thick unbound base layer are presented in Table 2. To allow water to pass through the unbound base layer, bottom-perforated moulds were used and to prevent fine particles being washed out, the bottom and sides were coated with geotextile. The test setup is presented in Figure 3. To create a paving system, pervious concrete flags were combined with unbound base layers whose properties are presented in Table 2 (Mould 1 and Mould 2). This way two different paving systems were created: paving system 1 (pervious concrete flags + Mould 1) and paving system 2 (pervious concrete flags + Mould 2).

**Figure 4:**
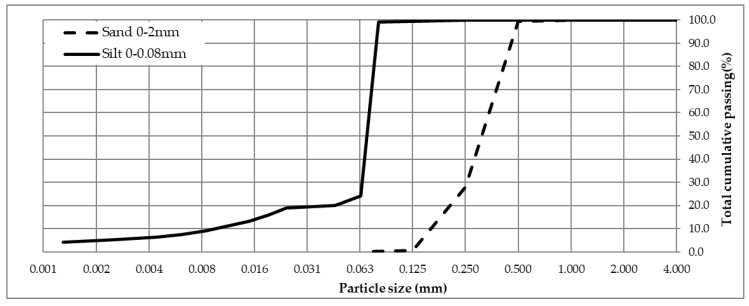
The grain size distribution of used clogging agent.

**Figure 5:**
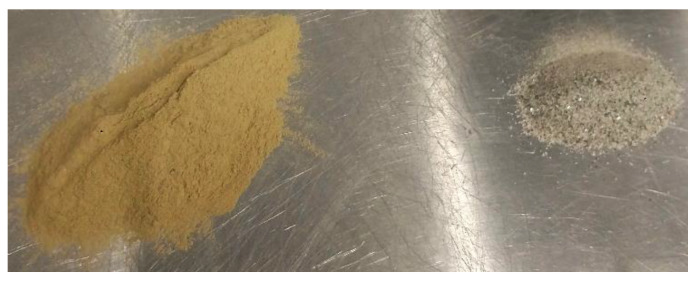
The appearance of used clogging agent: silt and sand.

For simulating clogging, a combination of silt (0–80 µm) and sand (0–2 mm) suspended in water were used in a ratio of 75–25%, respectively [32]. The grain size distribution of used silt was determined by a hydrometer and of used sand by sieving (Figure 4). For sand, there were 0.2% particles finer than 63 µm. According to [42], total suspended soil (TSS) concentration in highway runoff (urban highways with average traffic of more than 30,000 vehicles per day) is set to be 142 mg/L. To simulate accelerated clogging in extreme conditions, a 100-time higher concentration was used. Therefore, the clogging agent was 51 g of solid dissolved in 3.6 L of water, used in the prewetting procedure while implementing the ASTM C 1701-09 [15] procedure, without UBC simulation. Figure 5 presents the clogging agent used.

## 3. Results and Discussion

Presented results are the average value of three samples per mixture with the variation of the results within 10%.

### 3.1. Pervious Concrete Mechanical Properties

Results of the hardened concrete mechanical characteristics investigation are presented in Table 3. The mechanical characteristics of pervious concrete, depending on the aggregate type or grain size are well known and have been thoroughly researched. Discussion on the influence of dolomite, steel slag and diabase aggregate type and fraction size on mechanical characteristics are presented in detail in [16]. Within this research, mechanical characterization is used for evaluating potential applications of a particular mixture. Namely, compressive and flexural strengths are used as design criteria for concrete pavements, while porosity is a basic pervious concrete characteristic, influencing its drainage potential. In that sense, we discuss only peak values, potential application and enhancement of a particular mixture.

As presented in Table 3, maximal compressive and flexural strength is obtained for mixture M3, having only 4–8 mm fraction dolomite aggregate. The lowest values are obtained for mixture M10, having 90% of 4–8 mm and 10% of 0–2 mm fractions diabase aggregate. Results of mixtures M9 and M10 should be highlighted, as the only difference between them is in the type of fine fraction, i.e., sand or fine diabase. Using 10% sand instead of 10% fine fraction diabase (0–2 mm) results in a significant increase in compressive and flexural strength with negligible reduction in porosity. This is important for applications where higher strength is needed and a slightly lower infiltration rate acceptable.

Porosity is usually discussed in a view of infiltration rate so it will be discussed within Section 3.2. In terms of mechanical characterization, it can be observed that porosity is inversely proportional to obtained strengths, i.e., highest for M10 mixture and lowest for M3 mixture. Therefore, mixture M3 presents a potential for further strength enhancement e.g., through the addition of polymers, fibres, short-duration vibration during the installation, or simply adjusting the mix design procedure. Strength increase of this mixture could decrease porosity, but the obtained porosity of 19% is high enough to complement the minimum value of 11%.

### 3.2. Drainage Characteristics

Results of infiltration rate obtained on small cylindrical samples of uncontaminated pervious concrete by the falling head method (FHM), on pervious concrete flags before and after clogging, as well as on paving systems tested following ASTM C1701 are presented in Table 4.

The influence of clogging in pervious concrete flags expressed as a reduction in infiltration rate due to clogging (Table 5), is calculated as a difference between infiltration rates obtained on samples after and before clogging. The difference between infiltration rates in paving systems and pervious concrete flags before clogging, in Table 5, is designated as a reduction in infiltration rate due to mould properties (Mould 1 or Mould 2).

When the results obtained by two utilized methods, the falling head method (FHM) and method for inbuilding pervious concrete per ASTM C1701-09, are analysed it can be seen that the FHM results are higher (Table 4). On average for all tested mixtures, this equates to FHM results being 2.6 cm/s greater. However, results trends among all tested mixtures utilizing both methods are similar. Within both tests, the highest infiltration rate is obtained for mixture M11 (coarse diabase aggregate + 10% sand) and the lowest for mixture M3 (60% 4–8 mm fraction + 30% 8–16 mm fraction steel slag aggregate + 10% sand). When analysing the influence of the type of aggregate, diabase aggregate provides the highest infiltration rates, compared to dolomite and steel slag aggregate mixtures, which is attributed to uniform pore distribution [16]. Generally, the infiltration rate increases with the increase in porosity, but a strong correlation between these parameters cannot be defined. Porosity is here defined as the volumetric characteristic of tested pervious concrete samples, while infiltration rate also depends on pore connectivity and distribution within a tested sample. A similar conclusion is observed in [43].

**Figure 6:**
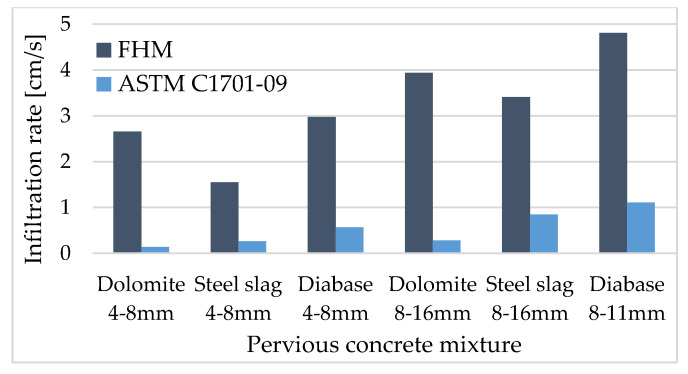
Results of infiltration rate for single-sized mixtures.

The pervious concrete mixtures with coarse aggregate have better permeability characteristics, regardless of aggregate type (Figure 6). This is in agreement with [44]; for mixtures having small aggregate grains, which also have small sectional areas in connected voids, the meandric path is formed for water to run through. For coarse grain aggregate mixtures, larger sectional area and straight paths for water are formed.

Different coarse to small fraction ratios were used to analyse in more detail how the variation of aggregate size influences pervious concrete characteristics, i.e., does it enhance strength characteristics while preserving porosity and permeability. For dolomite aggregate, as presented in Table 3 and Table 4, optimal porosity and infiltration rate, according to both methods, seem to be for the mixture having up to 30% of 4–8 mm aggregate fraction. For steel slag mixtures, this conclusion could not be derived—as stated earlier, porosity and infiltration rate are dependent on pore size and pore connectivity and, as presented in Figure 1, steel slag aggregate has a rough, porous surface which contributes to high variation in test results. This relates to a recommendation that the type of aggregate along with its surface characteristics should be taken into account when designing a mix composition.

**Figure 7:**
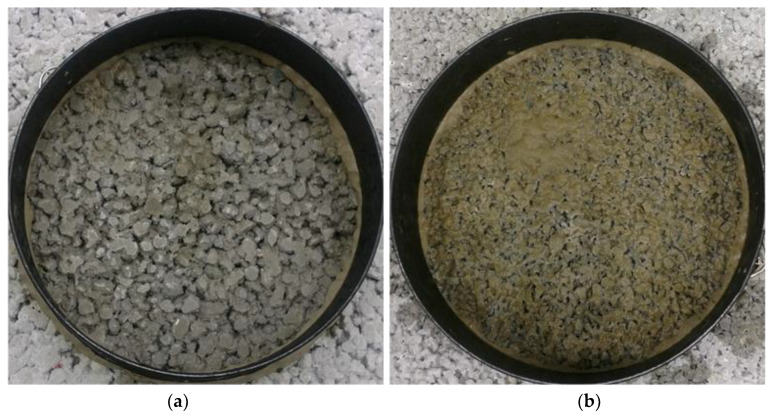
Sample surface after clogging test, (**a**) coarse- and (**b**) small-size aggregate mixtures.

As presented in Table 5, a reduction in infiltration rate due to clogging is observed as being between 1% for M11 and 27% for mixture M3. For mixtures with dolomite aggregate (M1 to M4), the highest reduction in infiltration rate is observed, between 6% and 27%. The lowest reductions are observed for mixtures with diabase aggregate (M9 to M11), being only 1% to 6%, which can be attributed to high porosity and particularly due to the presence of large pores at the surface. Figure 7 presents the surface of the samples after the clogging test for small-size and coarse-size aggregate mixtures.

**Figure 8:**
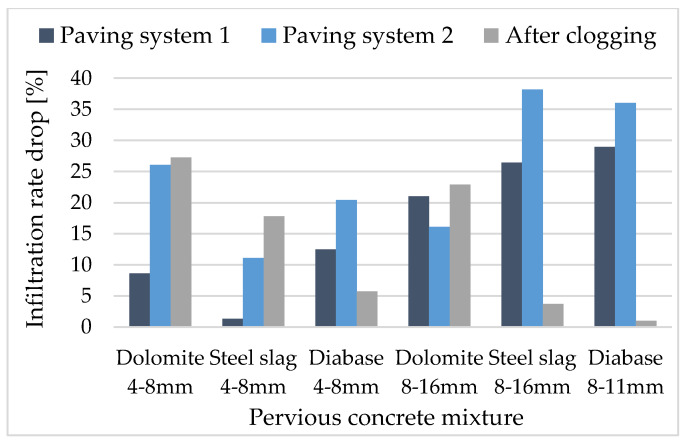
Results of an infiltration rate drop for single-sized mixtures.

Small-size aggregate mixtures have a higher infiltration rate reduction (Figure 8), primarily due to smaller surface pores which are blocked by the clogging material. The difference in the reduction of infiltration rate for small and coarse aggregate mixtures, for dolomite and diabase aggregates, is up to 5% (4.33% and 4.73% respectively), which is favourable and entails that a portion of small aggregate is desirable, particularly in wearing (surface) course, despite higher clogging infiltration rate drop. It should be pointed out that within this research, only one clogging event was undertaken, but with a significantly higher clogging agent concentration than it should appear in a pavement service life. For a holistic approach to pervious concrete pavement behavioural assessment in clogging conditions, repetitive clogging events could be tested with different clogging agent types and with a simulation of some kind of a maintenance program as presented in different literature [21,22,26,27,28].

**Figure 9:**
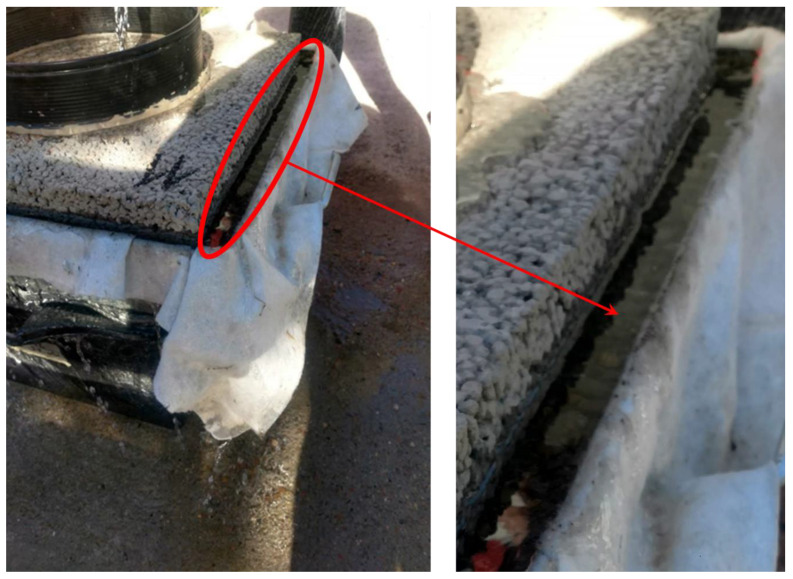
Water film as a result of a discrepancy in pervious concrete infiltration and unbound base course (UBC) exfiltration.

As presented in Figure 8, there is a significant infiltration rate drop when a real pavement system is simulated by the unbound base course (UBC) beneath the pervious concrete flag. The infiltration rate drop for UBC inbuilt in paving system 1 is less than that of UBC inbuild in paving system 2 due to 160 kg/m^3^ lower density, with the difference being 1% to 30%. The highest drops were observed for coarse aggregate mixtures, with up to 38% for M8, which indicates that the influence of UBC density is higher for coarse aggregate mixtures. For mixtures with higher porosity and large pores at the surface, water velocity is high and UBC exfiltration is insufficient, causing the creation of a water film at the contact of the pervious concrete slab and UBC (Figure 9). While coarse aggregate mixtures are less sensitive to clogging, subgrade exfiltration seems to be a flow-limiting element which is in accordance with findings presented in [31]. For small size aggregate mixtures, pervious concrete slab clogging and UBC exfiltration seems to have a similar influence on the pavement system. It should be pointed out that the mixture with a combination of small and coarse dolomite aggregate (M2) obtains a reduction in infiltration rate below 10% for all three scenarios (Table 5), less than all other dolomite aggregate mixtures. Finally, it is important to find a balance between pervious concrete infiltration and UBC exfiltration rate to reduce potential pavement damage and increase durability. An excessive pervious concrete infiltration rate could adversely influence the pavement system due to UBC inability of sufficient exfiltration. Therefore, when designing the pervious concrete system, all elements with its specific characteristics should be considered, particularly when designing coarse aggregate mixtures.

## 4. Conclusions

Drainage characteristics of 11 pervious concrete mixtures were investigated within this research to define a suitable mixture for low-traffic pavement surfaces. The aim was to define the influence of clogging and the impact of the unbound base layer on pervious concrete infiltration ratio reduction of a pavement surface course. Based on the results, the following conclusions can be drawn:

(1) Mechanical characteristics of pervious concrete can be significantly influenced by an educated selection of the type of fine fraction. Using 10% sand instead of 10% diabase of the fraction 0–2 mm results in a significant increase in compressive and flexural strength with negligible reduction in porosity.
(2) Pervious concrete mixtures with coarse aggregate show beneficial permeability characteristics, regardless of aggregate type.
(3) Optimal porosity and infiltration rate with dolomite aggregate can be achieved with a mixture having 30% of 4–8 mm aggregate fraction.
(4) Design of pervious concrete mixture composition should consider the type of used aggregate with its surface characteristics.
(5) Small-size aggregate mixtures of pervious concrete have higher infiltration rate reduction due to clogging. However, due to a small difference in infiltration rate drop for small and coarse aggregate mixtures and favourable influence on mechanical characteristics, using a portion of small aggregate is desirable, particularly in wearing (surface) course.
(6) A significant infiltration rate drop, particularly within coarse aggregate mixtures, occurs when a real pavement system is simulated by the unbound base course beneath the pervious concrete slab.
(7) Mixtures with a combination of small and coarse dolomite aggregate exhibit less than 10% infiltration rate drop under the influence of clogging or UBC. Generally, it is important to find a balance between pervious concrete infiltration and UBC exfiltration rate. An excessive pervious concrete infiltration rate could adversely influence the pavement system due to UBC inability of sufficient exfiltration, which could lead to pavement damage. Pervious concrete mixtures with a combination of small and coarse aggregate should be considered.

The conclusions presented here are drawn from the research conducted on a limited number of samples and, to reach general conclusions, more tests are to be conducted.

## Figures and Tables

**Table 1:** Pervious concrete mixture composition—fraction share in %.

Mixture	Sand	Dolomite		Steel Slag		Diabase		
	0–2 mm	4–8 mm	8–16 mm	4–8 mm	8–16 mm	0–2 mm	4–8 mm	8–11 mm
M1	10	60	30					
M2	10	30	60					
M3	−	100	−					
M4	−	−	100					
M5	10			60	30			
M6	10			30	60			
M7	−			100	−			
M8	−			−	100			
M9	10					−	90	−
M10	−					10	90	−
M11	10					−	−	90

**Table 2:** Characteristics of unbound base layer - reference material and inbuild in moulds.

Material	Dry Density (g/cm^3^)	Water Content (%)
Gravel	2.01	5.04
Mould 1 *	1.65	4.25
Mould 2 *	1.81	4.24

* gravel after compaction.

**Table 3:** Results of pervious concrete compressive strength (f_c_), flexural strength (f_v_) and porosity.

Mixture	f_c_ [MPa]	f_v_ [MPa]	Porosity (%)
M1	19.56	3.18	20.81
M2	17.80	2.23	25.33
M3	21.09	3.21	18.69
M4	14.27	2.51	21.00
M5	15.19	2.05	25.33
M6	13.15	1.96	28.70
M7	10.31	1.72	28.45
M8	15.80	1.59	23.75
M9	10.87	2.33	30.81
M10	8.17	1.49	32.35
M11	15.57	2.01	25.94

**Table 4:** Results of pervious concrete infiltration rate tests.

Mixture	Infiltration Rate by FHM, Uncontaminated Samples (cm/s)	Infiltration Rate by ASTM C1701, Before Clogging (cm/s)	Infiltration Rate by ASTM C1701, After Clogging (cm/s)	Infiltration Rate by ASTM C1701, Paving System 1 (cm/s)	Infiltration Rate by ASTM C1701, Paving System 2 (cm/s)
M1	2.057	0.130	0.095	0.125	0.108
M2	3.323	0.338	0.319	0.333	0.307
M3	2.659	0.138	0.100	0.126	0.102
M4	3.938	0.349	0.269	0.275	0.292
M5	1.488	0.086	0.067	0.085	0.059
M6	2.832	0.110	0.082	0.099	0.094
M7	1.546	0.260	0.214	0.256	0.231
M8	3.409	0.849	0.817	0.624	0.525
M9	2.974	0.566	0.534	0.495	0.450
M10	3.757	0.542	0.508	0.542	0.539
M11	4.810	1.107	1.096	0.787	0.708

**Table 5:** Reduction in infiltration rates due to clogging and mould properties.

Mixture	Reduction in Infiltration Rate Due to Clogging (%)	Reduction in Infiltration Rate Due to Mould 1 (%)	Reduction in Infiltration Rate Due to Mould 2 (%)
M1	26.78	4.05	17.20
M2	5.71	1.53	9.24
M3	27.23	8.64	26.03
M4	22.90	21.03	16.13
M5	21.28	0.87	30.51
M6	25.91	9.74	14.93
M7	17.83	1.33	11.09
M8	3.72	26.43	38.17
M9	5.72	12.49	20.45
M10	6.26	0.00	0.55
M11	0.99	28.92	36.02
